# Nonparametric Simulation of Signal Transduction Networks with Semi-Synchronized Update

**DOI:** 10.1371/journal.pone.0039643

**Published:** 2012-06-21

**Authors:** Isar Nassiri, Ali Masoudi-Nejad, Mahdi Jalili, Ali Moeini

**Affiliations:** 1 Laboratory of System Biology and Bioinformatics (LBB), Institute of Biochemistry and Biophysics, University of Tehran, Tehran, Iran; 2 Department of Computer Engineering, Sharif University of Technology, Tehran, Iran; 3 Department of Algorithms and Computation, College of Engineering, University of Tehran, Tehran, Iran; Semmelweis University, Hungary

## Abstract

Simulating signal transduction in cellular signaling networks provides predictions of network dynamics by quantifying the changes in concentration and activity-level of the individual proteins. Since numerical values of kinetic parameters might be difficult to obtain, it is imperative to develop non-parametric approaches that combine the connectivity of a network with the response of individual proteins to signals which travel through the network. The activity levels of signaling proteins computed through existing non-parametric modeling tools do not show significant correlations with the observed values in experimental results. In this work we developed a non-parametric computational framework to describe the profile of the evolving process and the time course of the proportion of active form of molecules in the signal transduction networks. The model is also capable of incorporating perturbations. The model was validated on four signaling networks showing that it can effectively uncover the activity levels and trends of response during signal transduction process.

## Introduction

Cells sense, monitor and process signals originating from the environment. Monitoring external conditions requires the signal transduction to the site of action and thereby triggers various biological responses. Cells have developed signal transduction pathways, which facilitate signal transmission from the receptors to the target molecules by cascades of modifications to cellular molecules such as phosphorylation [1]. Evolving proteomic approaches to network biology have focused on measuring the changes in abundances of signaling proteins in active forms (*e.g.* phosphorylated form) under different experimental conditions [2]. Examples of such protein-based datasets becoming more frequent in the literature [3], [4], [5], [6], [7]. At the present, this type of studies on protein functional status focuses on frequent sampling of a limited number of key molecules [4], [8]. This necessitates developing mathematical methods to prioritize selection of the molecules such that measuring their activity to be informative and capable of predicting the outcome of new experiments [9], [10].

Evidently, it is important to apply adequate updating rules to choose biologically correct model [11]. During the signal transduction process, always time delays are associated with the transport of signaling molecules to reach the site of action [12]. For example, the transport of signaling molecules over intracellular distances of more than a few micrometers normally requires facilitated transport mechanisms such as movement of phosphorylated kinases on the scaffolds [13], [14], [15]. The other issue is the stochasticity in signaling processes at the molecular level. The origin of stochasticity can be due to chaotic births and deaths of individual molecules as well as the extracellular noise due to environmental fluctuations [16]. Such delays and stochastic noises are source of inherent fluctuations in transferred signal [17]. Generally, asynchronous and continuous models, in which all edges demonstrate different efficiencies for making signal transition, are closer to biological phenomena [18].

A number of studies have shown close relation between structure of biological networks and their functional phenotypes [19], [20]. Therefore, scholars struggled to develop nonparametric methods that are based on only network topology [9], [10], [21], [22]. However, no model has been reported to compute both the proportion of active form and the trend of activation of given molecule in signal transduction networks [9], [10]. The present study proposes an efficient nonparametric method for accurate identification of activity trends and the proportion of active form for each member of the signaling networks. We validated our approach for logical modeling on four signaling networks. We show that activity level and activation trend of signaling proteins observed through the proposed model have significant correlations with experimental results.

## Materials and Methods

Signaling networks are represented as directed graphs where nodes denote signaling components and edges represent the direction of information flow. Edges are labeled with positive (+1) or negative (−1) signs, which define activation or inhibition, respectively. The input (source) nodes represent the ligands or their receptors, the intermediate nodes consist of various kinases and second messengers, and the output (sink) nodes represent transcription factors, channels, cytoskeleton, motility components, or cellular responses [23]. Through the interactions in the network, signals propagate from the receptor (source node) to the downstream and target molecules (sink nodes).

The proposed dynamic model follows the changes in the activity levels of signaling proteins (between 0 and 1) in time steps and the way that signals propagate through molecular interactions [24]. Thus, in some sense, the predictions returned by our simulator can be interpreted like the normalized results of bead-based micro-ELISA assay [4], [25]. In the following sections, we describe different parts of our method to simulate signaling networks.

### Edge weighting

**Figure 1:**
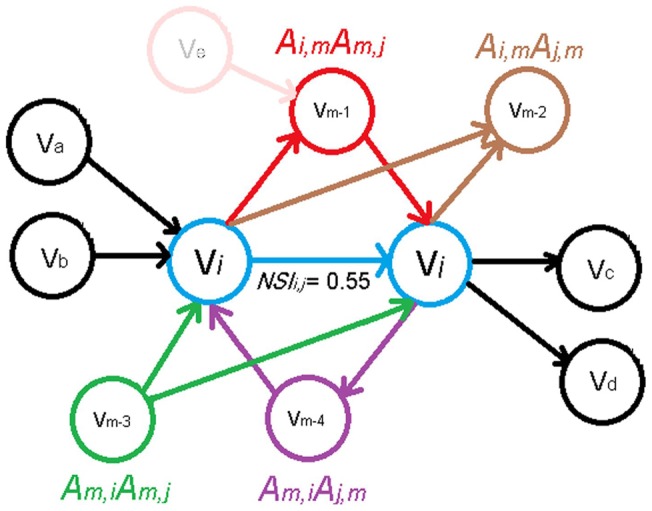
Weighting of the edge by Normalize SimilarityIndex (NSI). 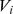
 and 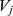 are connected with one directed edge (C_ij_ = 1). Node 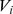 and 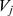 are also connected with four common node (V_m−1_,V_m−2_,V_m−3_,V_m−4_), and in total with eight distinct neighbors (V_m−1_,V_m−2_,V_m−3_,V_m−4_,V_a_, V_b_, V_c_, V_d_). The NSI will then be NSI_ij_  = 5/9 = 0.55.

Matching connection is a measure used to establish similarity between two nodes (proteins) [26], [27]. If nodes *i* and *j* have a number of common neighbors, they may be related to each other, even though they are not directly connected [28], [29], [30]. Here in order to quantify the efficiency of signal transduction between the two nodes adjacent to the given edge, we used similarity index and took into account the existence of matching connections. The extension is called Normalized Similarity Index (NSI). Let us consider a directed network with adjacency matrix *A*, and *n* nodes that are denoted from 1 to *n*. Let there also be a *n×n* similarity matrix **S** =  (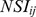) satisfying 0<
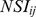
≤1, 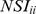 = 0, and 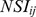  =  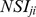 for all *i*, *j* ∈ {1, …,*n*}. The NSI of edge between nodes *i* and *j* is computed considering the number of matching connections of *i* and *j* to any other node *m* 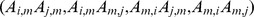, and the number of connections (edges) between the node *i* and *j* (*C_ij_*). More precisely,
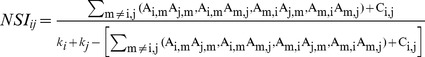
(1)where *k_i_* is degree of node *i*. Figure 1 shows a graphics for computing NSI. Each edge is associated with a weight (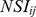) ranging from 0 to 1 that represents the efficiency of signals passing along the edge. Edge weighting by NSI suggests that inside the highly connected parts of network, the efficiency of signal propagation along the edges is mainly, due to the facility posed by high NSI values. Data from previous studies supported the idea that cellular signals are transmitted dominantly through pathways of highly connected proteins [31].

The similarity index is often used to cluster different components of biological networks that are functionally similar [32], [33]. The definition draws upon the notion that two molecules (nodes) could be considered similar if they perceive the rest of the nodes within the network in a similar way [26], [27], [34]. Previous similarity indices are based on the number of common neighbors shared by two non-adjacent nodes *i* and *j* [28], [32], [33], [34]. However, NSI quantifies the similarity between two connected nodes. The VOSveiwer program was used for visulization of weighted networks by similarity measure [35].

### Simulation of signal transduction and weighting the nodes

The simulation starts from signal ligand and iteratively traverses the whole network by a breadth-first-search (BFS). As we are only interested in the connected sub-network that begins with the first node, the nodes that are not reachable from the first node are simply ignored. The state of a node *j*, (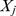), is defined as a proportion of molecules in active form (*e.g.* phosphorelated) and gets a value between 0 and 1. At each time interval *t*, the *X*(*t*) is changed or updated based on its value in the previous time step, 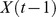, the statuses of the set of activator 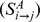, and those of repressor nodes feeding into it 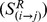 according to the following formula:
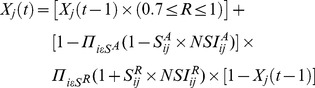
(2)where, each directed edge (*i, j*) models the transduction of signals from node *i* to node *j* of activation (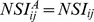) or repressor (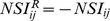) type. In order to explain clearly the equation 2, let *X  =  (X_A_, X_I_)*  =  ({#Active form}, {#Inactive form}) be the states of a given molecule at time *t*. If *P*(*X_A_, t*) is the probability function for the state ({#Active form})  =  *X_A_* at time *t*, in order to simplifying the expression we can use *P*(*X_A_*, *t*)  =  *X*(*t*). Therefore, *X*(*t*) is the probability or proportion of the active form of given molecule at time *t*.

Although we know a great deal about global protein stability profiling, little is known about global stability of signaling proteins in the active form (e.g. phosphorylated) [36], [37]. In the proposed model, the relative stability (R) of proteins in active form is chosen randomly and changed after each time step 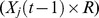 [20]. We adopted equation 2 according to the method proposed by *Van Kampen* for selecting the subset of molecules in the active form in preceding iteration to be passed to next iteration [38]. First, we developed a deterministic equations of the experimental system, and then added a noise term (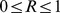), and adjusted a specified random function to reproduce a correct mean as taken from the experimental results (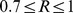). The noise term is independent and uncorrelated with the time span of the system. We used [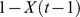] in formula to ensure that the activation of a node has bounded in the interval [0–1], regardless of the number of iterations.

In the model, time is quantized into regular intervals (time-steps) as the longest duration required for state of all components to be updated [39]. We extended this basic model to account for variability in the duration of signal transmission by performing signal transduction to some nodes in an asynchronous update fashion, called semi-synchronous update [20], [21], [40]. If a directed network is imaged in hierarchical layout, in semi-synchronous update, each node in layer *d* at time-step *t* receive signals from its regulators in layer *d* and layer *d*-1 in *t*, and its regulators in downstream of layer *d* in *t*+1. Therefore, the state of a node in each time step depends on the signals transmitted to the nodes at time-step *t* or *t-1.* The rules for semi-synchronous algorithm can be written as:
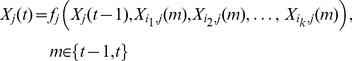
(3)where 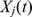 is the state of node *j* at time-step *t*, 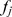 is the function associated with state of node *j* at time-step *t*-1 (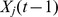) and its regulators (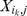) in previous time step (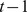) or current round of updates *t*.

Equation 2 is derived from a specific form of non-linear dynamic representation of the regulatory networks of transcription. It is assumed that all transcription factors independently bind at the available distinct sites of promoter region without interacting with each other [41]. In updating the nodes, we also assumed that the order of incoming signals does not affect the outcome, i.e., 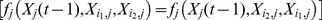 in Equation (3).

In the first step of simulation, user can specify the activity of first node (proportion of active form) as a source of signal. The activity of first node (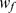) changes systematically according to the following relation:
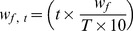
(4)where, *T* is a user-specified number of iterations, 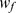 is a user-specified activity of first node, and 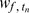 is the weight of first node in iteration *t*. The term 10 in the denominator confines the proportion of receptors in active form (binds its ligand), that is less than 1, because almost 10% of the total receptors pool are usually in high-affinity form [42], [43]. The activities of other nodes are initially equal to 0.

As the main focus of this study was designing a purely non-parametric simulation method, we used network topology to predict the half-lives of proteins. Wang *et al.* (2009) systematically examined the entire human signaling network and mapped the enrichment of proteins with different half-lives in the groups of nodes that were categorized based on their degree in network and cellular location [44]. According to their results, 77.5% of the nodes with degree equal to or bigger than 6, 73.6% of nuclear proteins, and 48.9% of ligands had long half-lives. Therefore, for the nodes with a degree equal to or bigger than 6, and out-degree (sink nodes) or in-degree (source nodes) equal to 0, we defined two kinds of proportion of activity: 1- if node has short half-life and 2- if it has long half-life [44], [45].

### Simulation of perturbation experiments

**Figure 2:**
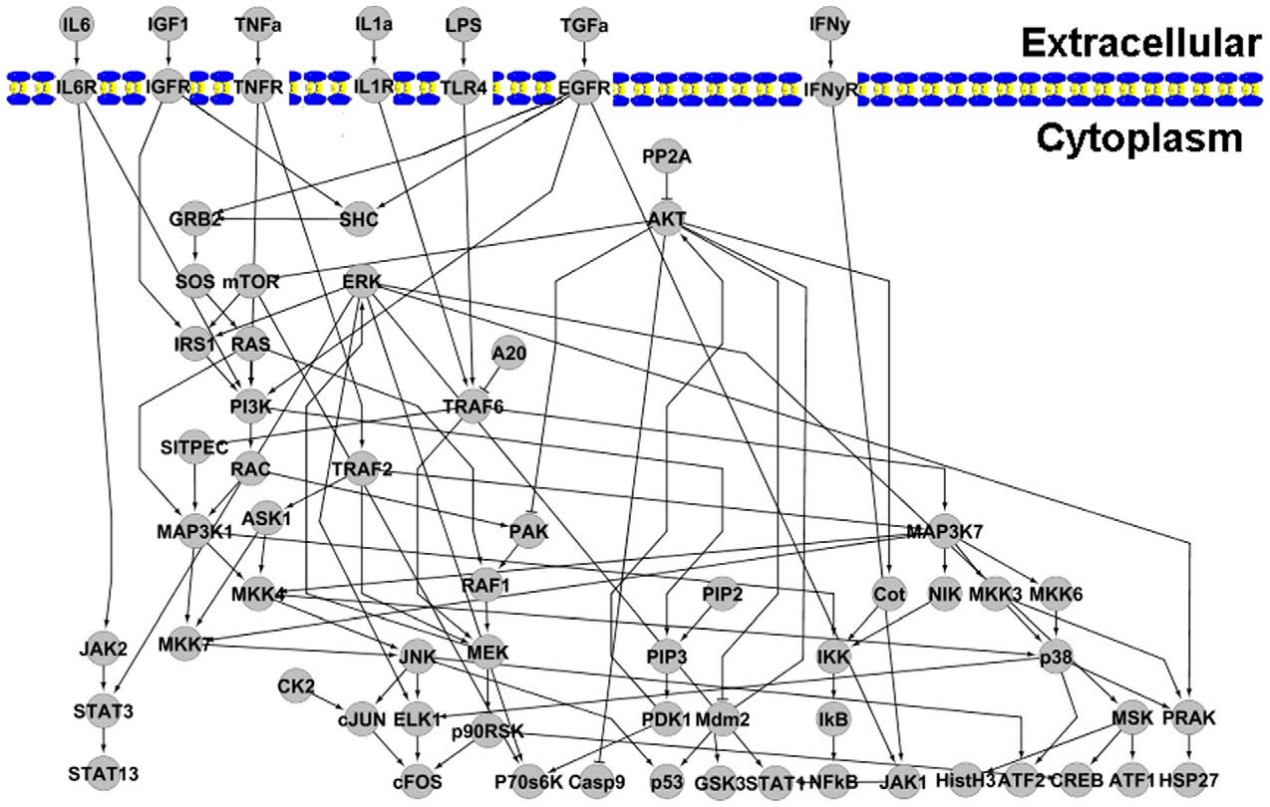
Inflammatory and growth signaling network in human hepatocyte cells. Activator (→) and inhibitor (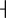) reactions are indicated as edges. Nodes represented in the extracellular space are ligands.

**Figure 3:**
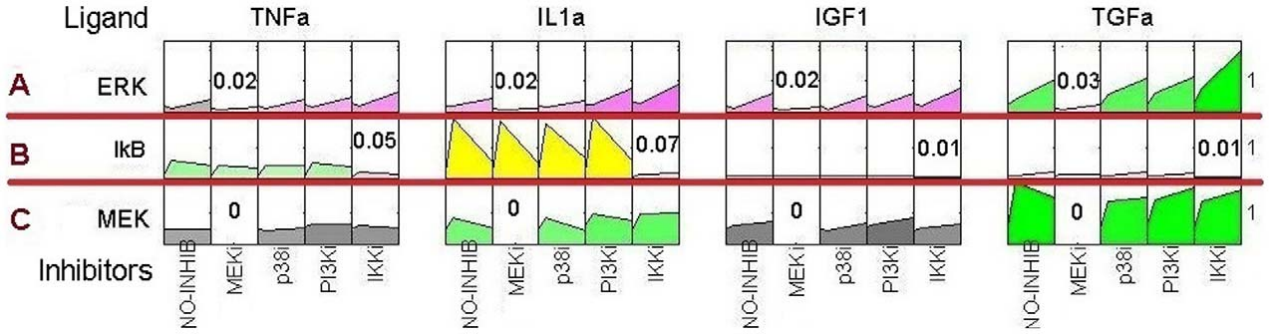
Signaling dataset of three proteins from human hepatocyte cells. Rows represent the measures of three assayed intracellular molecules, and columns represent 4 different ligands. For each combination of ligand and target molecule, one of 4 kinase inhibitors was applied, as indicated in the schematic diagram below the data. The numbers on the right of the figure indicate the maximum values of the corresponding row. The Figure was created using Data-Rail program [4], [48].

Typically, the functional importance of a molecule in signal transduction network is determined by cell response after its inhibition by specific chemical inhibitors or interference with the siRNA [4]. In many existing methods, molecular inhibition is simulated by deleting the corresponding nodes and edges incident on it [46], [47]. However, the disruption of any molecule in cell signaling network may lead to a cascade breakdown of other downstream molecules. We briefly considered some examples from the perturbation results of the phosphoproteins in the inflammatory and growth signaling network in human hepatoblastoma cells [4]. Figure 2 depicts the inflammatory and growth signaling network in human hepatocyte cells. The experimental results are plotted in Figure 3, which is organized into panels, corresponding to the various ligands [4], [48]. If we simulate molecular inhibition of MEK and IKK by deleting the corresponding nodes and the edges incident on them, the signal flow to the ERK and IκB is broken (Figure 2). However, according to the experimental results, treatment of MEK and IKK with their corresponding inhibitors and the measurements of the activity of their downstream proteins (ERK and IκB) have shown that a little signal flow can pass through the inhibited node, and the signals sent by upstream nodes can have inductive effect on its target molecules (Figure 3A and 3B) [4].

To take into account the gain of function or inhibitory effects on the molecule(s), we used a complementary node for target node [39], [49], [50]. To simulate the influence of the inhibitory effect on the signaling component, target node receives constant inhibitory messages from the complementary node during simulation; however, signal flow can pass through the inhibited node in some iteration. When the measured molecule is inhibited (*e.g.* MEK), its measurement cannot be used in experimental studies (Figure 3C) [4]. Therefore, in our method the proportion of active form of inhibited node is denoted with the input value specified by the user (often 0).

### Overview of the tool

The Nonparametric Simulator of Signaling Networks (NSIN) program was written in C^++^, and is freely available at http://lbb.ut.ac.ir/Download/LBBsoft/NSIN. The inputs to NSIN are text files specifying a directed graph in which each node represents a specific molecule (Input A), and each directed edge models the transduction of signals of activation (+1) or blockage (−1) type (Input B). The simulator provides both single and set of running modes. In single mode, users can specify the activity of the input node (between 0 and 1), while the activities of the other nodes change as a function of initial stimulus. A set-mode run, consists of multiple inputs (up to 10), each with the same or different activity. The simulation stops after a number of iterations specified by the user. As output, the program provides a set of continues values for proportion of molecules in active form, and the weight of edges in the network according to the NSI formula. For some nodes, two kinds of proportion of activity including short 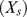 and long half-lives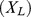, are defined. In addition, the in-degree and out-degree of each node is specified and can be used as an indicator to select the proper half-lives for molecules.

### Validating the model

Experiments for model validation can be divided into three major types. The first type involves stimulation of system with a step change in the input and measuring the changes in activities of various downstream species as a function of time [9]. Second type of experiment for model validation involves changes in the input by adding a high concentration of ligand and then measuring activity changes of various downstream species in several intervals [51]. Third type is used to provide insight of molecular activity trends in response to the external stimulus and perturbation [10]. We validated our model on four signaling networks including the EGFR/IGF-1R/IR (type I experiment for model validation), inflammatory and growth signaling networks in human hepatocyte cells (type II), and MAPK1,2 and AKT signaling network downstream from EGFR in MDA231 breast cancer cell line (type III). We also used human signaling network (containing 1634 nodes and 5089 edges), and mouse hippocampal CA1 neuron network (containing 545 nodes and 1259 edges) for visualization of organization in signal transduction networks weighted by similarity measure [31], [52].

### Signaling network of EGFR/IGF-1R/IR

Zielinski *et* *al.* (2009) assembled canonical pathways, downstream the three major receptors, including insulin receptor (IR), IGF-1R, and EGFR in SKOV3 cell line [9]. The network has 82 nodes and 128 edges, of which EGF, IGF, and insulin can be considered as the input nodes (Table S1).

### Signaling networks of human hepatocyte cells

We obtained the connectivity for two signaling networks of human hepatocyte cells from published literatures (Table S2 and S3) [53], [54]. Network of inflammatory and growth signaling is depicted in Figure 2.

### MAPK1,2 and AKT signaling network downstream from EGFR in breast cancer cell line

We obtained the connectivity MAPK1,2 and AKT signaling network downstream from EGFR in MDA231 breast cancer cell line from a published literature [10]. There is a gain of function mutation in Ras for the MDA231 cell line [10].

## Results

**Figure 4:**
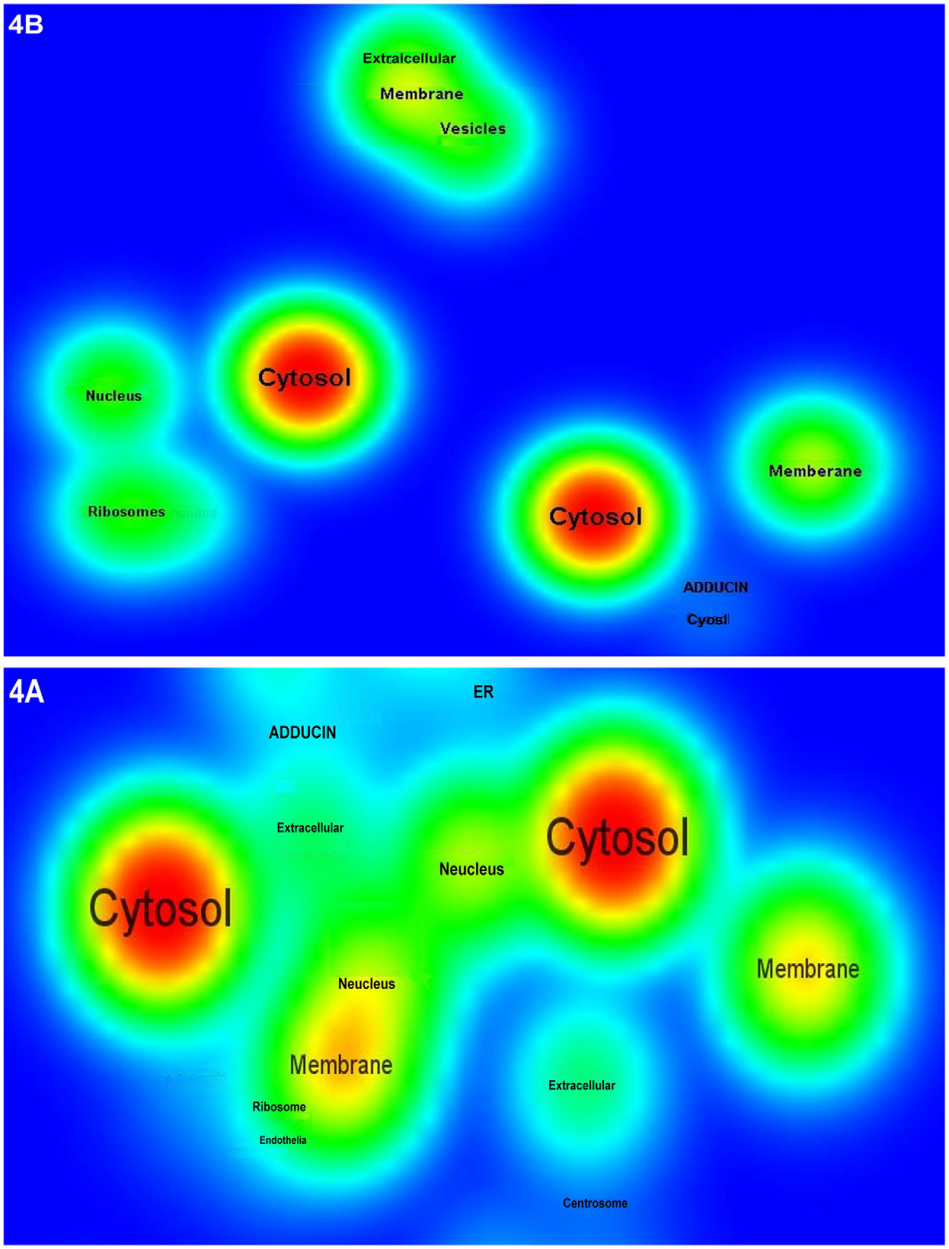
Cluster density view of the weighted cellular signaling networks by applying similarity measure. (A) human cancer cell signaling, and (B) mouse hippocampus CA1 neural networks were weighted by similarity measure. Nodes were labeled according to their position in the cell, including cell membrane, adducin, cell adhesion, centrosome, cytoskeleton, endothelial, endoplasmic reticulum, cytosolic, extracellular space, golgi apparatus, lysosome, mitochondria, nucleus, ribosome and vesicles. VOSveiwer program was used for visualizing connectivity-based clustering patterns [35]. This tool provides visualization of similarities, where objects with high similarity are located close to each other and those with low similarity are located far from each other.

In this study, we developed a computational framework to model the concentration of the active form of given proteins and obtained the quantities of proteins involved in cell-specific signaling networks. In the proposed modeling method the weight of a node was the product of its activity at time *t*-1, the amounts of signals received by upstream nodes and the weight of activating or blocking edges. We calculated the edge weights through the NSI formula (see Methods), as the efficiency of transmission signal between the nodes. As the signal transduction network was given appropriate weights using the similarity measure, cluster patterns became visible (Figure 4A and 4B). The cluster patterns corresponded to different parts of eukaryotic cells including cell membrane, cytoplasm and nucleus. These profiles suggest that inside the clusters, the efficiency of signal propagation along the edges is more than their outside that is primarily due to the facility posed by high NSI values.

### Case studies for model validation

We chose four signaling networks as the EGFR/IGF-1R/IR, inflammatory and growth signals in human hepatocyte cells, and MAPK1,2 and AKT signaling network downstream from EGFR as the benchmarks for validating our method [9], [10], [53].

### Signaling network of EGFR/IGF-1R/IR

The ability of the model to predict the proportion of active molecules in response to different levels of stimulation was the first question that we considered. Since the output of the model is continuous, it is possible to evaluate the activation of target molecules in response to different concentrations of ligands.

**Figure 5:**
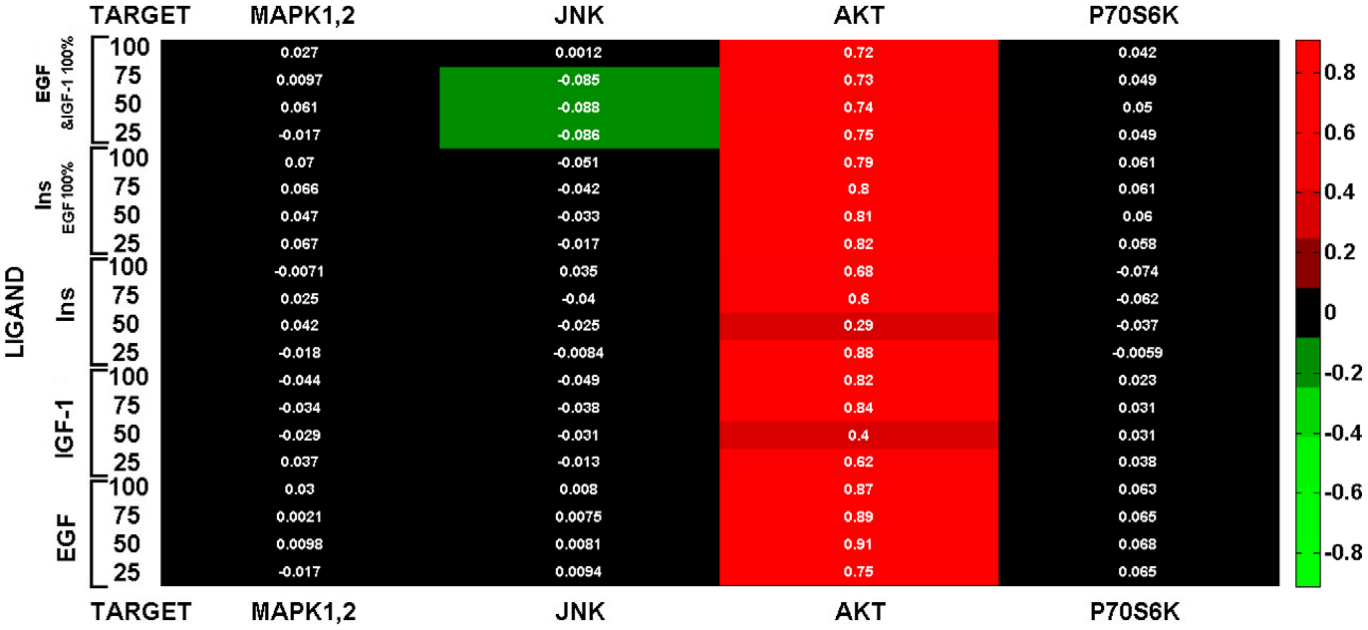
Comparison between the experimental results with those obtained through simulation. The activity level of four signaling molecules (MAPK1,2, JNK, AKT, and p70s6K) proteins in EGF/IGF/Insulin cell signaling network were simulated under activation of three ligand (EGF, insulin, and IGF-1) at 100 iterations. If the value of difference between the experimental and simulation data is bigger than zero, the corresponding box is colored in red; if best agreement, the box is black; but if the value of the difference between simulation and experimental data be smaller than zero, the box is green.

We used the network constructed by combining EGF, IGF and insulin signaling pathways and related experimental results including the responses of selected molecules to the specific stimuli [9]. Four proteins with network crosstalk, ERK, AKT, p70s6K, and JNK, were selected. In order to compare the experimental results with those obtained through computations, four sets of simulations were performed by 25% step-wise increase in the receptor activation [9]. This set of simulations made possible to have translation of the molecular response into activation level. The experimental and simulation results are reported as proportion of each examined molecule in active form. According to the results, our model was in line with experimentally observed values with a Pearson correlation of 0.742 (*P*<10^−15^) (Figure 5) (Table S1).

We observed that computer simulation was able to recapitulate all the trends observed in the experimental studies. As expected, co-stimulation of the signaling with insulin and EGF led to the activation of JNK and MAPK1,2. Also, the activation of insulin and IGF-1 was translated to the activation of p70s6K during stimulation (Table S1) [9].

As mentioned before, our simulation method does not need experimental data such as the reaction rates or association constants. The performance of our method depends on how the underlying network is completed. For example, the discrepancy between the predictions of proportion of AKT molecules in active form with the experimental results, in almost all cases, can reveal that our information about the regulation of AKT in SKOV3 cell line was incomplete (Figure 5) (Table S1).

### Signaling networks of inflammatory and growth signals in hepatocyte cells

We further evaluated the ability of the model in predicting the outcome of experiments by using two datasets consisting of the activity levels of signaling proteins of primary and transformed hepatocytes (Huh7) under various perturbations [4], [7].

Inflammatory and growth signaling network in human hepatocyte cells included the pathways downstream the seven receptors, including IL6R, IGFR, TNFR, IL1R, TLR4, EGFR, and IFNγR (Figure 2). We simulated the activity levels of 14 signaling proteins (AKT, CREB, ERK, GSK3, HistH3, HSP27, IκB, IRS1s, JNK, MEK, p38, p70s6K, p90 & STAT3) under activation of IL1α, TGFα, IL6, TNFα and perturbations by two inhibitors (MEKi & PI3Ki) for all 168 possible pair-wise combinations at 100 iterations. Our tool showed that, predictions of activity levels of phosphoproteins under various perturbations agreed with those obtained experimentally with a Pearson correlation of 0.831 (*P*<10^−44^) (Table S2). We simulated the activation state of CREB, ERK, HistH3, HSP27, p90, IκB, JNK, MEK, p38, and p70s6 proteins in human hepatocyte cells under activation of IL1b, TGFa, HER, INS, TNFα and perturbations by cMETi, MEKi and PI3Ki for all 200 possible pair-wise combinations at 100 iterations. Simulation of activity levels of 10 signaling proteins under various perturbations agreed with experimental data (Pearson correlation of 0.817; P<10^−46^) (Table S3).

### MAPK1,2 and AKT signaling network downstream from EGFR

We tested the accuracy and performance of our method to simulate the effects of targeted manipulation in MAPK1,2 and AKT signaling network in MDA231 breast cancer cell line [10]. In the MDA231 cell line, there is a gain of function mutation in Ras. This is modeled using fixed activity assignments on Ras during the simulation [10].

We compared the simulation and experimental results through computing the proportion of active form of mTOR, GSK3β, p70s6K, AKT, and MAPK1,2 molecules under activation of EGF and inhibition of TSC2 [10]. Simulation of normal and perturbed signal transduction was performed in 100 iterations. The Wilcoxon test was used to study changes in the signal propagation between the simulation results before and after perturbation. The results produced through the simulation agreed with those obtained through the experiments (Table 1). We did not expect that the TSC2 perturbation can have a significant effect on the activity of GSK3β and AKT, which was what the statistical test indicated (Table 1) [10]. The mTOR, p70S6K, and MAPK1,2 showed a significant response to the perturbation, and the changes in mean activity were beyond the significance level of 0.01.

**Table 1:** The comparison between the simulation and experimental results of five signaling molecules under activation of EGF and inhibition of TSC2.

Molecule	Simulation Results		Change in Activity After TSC2 Inhibition		Wilcoxon-test	
	Normal	TSC2 inhibited	Experiment	Simulation	Z	P-value
mTOR	0.0038	0.022	↑	↑	−8.68	<10^−18^
MAPK1,2	0.017	0.014	↓ or -	↓	−4.21	<10^−5^
p70s6K	0.029	0.035	↑	↑	−8.68	<10^−18^
AKT	0.072	0.071	↓ or -	-	−1.26	0.206
GSK3β	0.025	0.026	-	-	−3.14	0.016

The P-values were calculated by Wilcoxon test and used to test the changes in simulation results after perturbation. The upward arrow (↑) indicates that the perturbation caused a rise in the proportion of molecules in active form; the straight line (-) indicates no change, and the downward arrow (↓) indicates decrease in the proportion of molecules in active form.

### Predictions made by the model

Activation of a T cell by exposure to specific agonist may lead to cytotoxic attacks on target cells, cytokine production or cell proliferation [55]. The process of T cell activation can be separated into a hierarchy of thresholds. In general, a hierarchy of thresholds is observed for T cell responses with the relative threshold order: S_cytotoxicity_ << S_cytokine production_ < S_cell proliferation_ (where S stands for potency of stimulus) [56]. These thresholds depend on the stimulus conditions with the most significant changes occurring in the presence of co-stimulation of receptors [57], [58]. The final level of this signaling process is the integration of signals to regulation of the gene transcription [59]. This process has been studied for its possible role in the diseases such as of autoimmune disorders, atopic dermatitis and fibrotic diseases [60], [61]. In order to have an example of how our modeling approach might be used for novel predictions, we used threshold concept in T cell activation problem.

We considered activation model motivated by the dependence of cell responses on the potency of the signal and simulated the effects of different treatments on NF-κB activation (Figure 2) [62]. Table 2 shows the results of simulating proportion of NF-κB molecules in active form were analyzed under combined and only treatment effects of five ligands including: IL1α, IL6, TNFα, IGF1, and TGFα. For each treatment condition, we simulated 100 time-steps. With respect to simulation results, NF-κB molecules appear to respond stronger under IL1α & IL6, IL1α & TNFα, IL1α & IGF1, and IL1α & TGFα treatments (Table 2). Hence, co-stimulation with IL1α may sufficiently stimulate the T cell to reach the proliferation threshold in response to treatment, as well as suggest effective target to perturb the progression of T cells to proliferation phase.

**Table 2:** Proportion of NF-κB molecules in active form under combined and only treatment effects of five ligands.

Signal		IL1α	IL6	TNFα	IGF1	TGFα
**IL1α**	**NF-kβ**	0.17				
**IL6**		0.26	0.10			
**TNFα**		0.25	0.18	0.18		
**IGF1**		0.26	0.22	0.24	0.20	
**TGFα**		0.25	0.20	0.24	0.23	0.18

The rows and columns of the table correspond to the treatment groups, and cells to proportion of NF-κB molecules in active form.

## Discussion

Modeling function of cellular networks in a dynamic fashion provides an optimal basis for elaborate study of cellular signal transduction [63]. However, lack of detailed concentration and kinetic data may make it difficult to use some modeling methods [64]. Thus, nonparametric modeling provides an alternative modeling approach to test hypothetical signaling networks [20], [65]. In this work we developed a non-parametric computational framework to describe the proportion of active form and the trend of activation of given molecules in signaling network. In the previous nonparametric methods, simulation has been performed in such a way that all the components changed their states simultaneously in a unit of time based on the assumption that every reaction in the network takes exactly single unit of time in the signaling process [9], [10]. Derek *et al.* (2008) presented a non-parametric and a Petri net-based model of cellular signaling networks [10]. This method provides insights into the trends of molecular activity-levels in response to an external stimulus, based on the network’s connectivity. Despite this success, the model could not predict the proportion of molecules in active form [10]. Zielinski *et al.* (2009) developed a network-specific model for dynamic simulation of signal transduction, and tested it on a network constructed by combining EGF, IGF and insulin signaling pathways [9]. The model agreed with many of the experimentally observed trends; however, it was notable to recapitulate the proportion of molecules in active form with significant correlation to the observed values in the experimental study [9]. In our proposed method, the defining dynamics occurred at the level of interactions among molecules, and coarse properties were computed by aggregating local quantities. This approach provides access to the microscopic dynamics which is hindered by the complexity of the system. The result was a fast method, which can provide insights into the proportion of molecules in active form and trends of molecular activity level in response to external stimuli. In our simulation method, node values were produced by combining two processes including edge weighting and simulation of signal flow from the initial node(s). During simulation, we used three updating strategies to reduce the artifacts due to the assumption of uniformity in reactions arising from synchronous updating methods. First, we employed a semi-synchronous updating scheme [40]. In our method, some of the incoming messages influence the updating state immediately, while others took longer to affect. Second, the level of activity for each node was transferring to next time-step with efficiency less than 1. This allowed us to take into account the relative stability of proteins in active form. Third, we used a specific function to weight the network edges and calibrated their efficiency for signal transition in the network. The outcome of the procedure used for edge weighting was a fractioned network to several clusters. These clusters were grouping molecules with the same place in the cell (*e.g.* nucleus). By coupling edge weighting with the statistical methodology of node weighting, we obtained a method capable of characterizing dynamic properties of signaling networks while using only network's connectivity information. Validation on several signaling networks showed that our method could effectively simulate both inhibited and constitutive activation of signal transduction components. Our simulation results were in strong agreements with the experimental results. Therefore, the present simulation method not only reproduces experimental data but also can predict non-intuitive and previously unknown responses. Also, the simulation results are capable of linking signal transduction to any type of quantifiable cellular responses such as cell growth, survival, apoptosis, necrosis, cytokine secretion, or transcriptional activity.

Our principal conclusion is that the dynamical phenotype possesses the ability of design according to the network topology. This finding corresponds conceptually to proteins where a two- and three-dimensional structure usually possesses design abilities according to the sequence of amino acids [66]. Our investigation showed that the dynamical phenotypes arise via the presence of conserved network links and could reflect wide variations in the level of activity at different positions. In summary, with the networks involved in case studies, our approach has proved itself as a promising tool to analyze signal transduction, effect of drugs and network modifications.

## Supporting Information

Table S1 **Simulation results of activity levels of four signaling proteins in signaling network of EGFR/IGF-1R/IR under various perturbations with comparison with experimental results.** (A) The proportion of MAPK1,2, JNK, AKT, and p70s6K molecules in active forms in response to the activation of EGFR, IGF-1R, and IR. (B) Activation of MAPK1,2, JNK, AKT, and p70s6K in response to co-stimulation of EGFR/IR and IGF-1R/EGFR/IR.
(XLSX)

Table S2 **The simulation results of activity levels of 14 signaling proteins in human hepatocyte cells under various perturbations, in comparison with experimental results.**
(XLSX)

Table S3 **The simulation results of activity levels of 10 signaling proteins in the Huh7 cell line under various perturbations, in comparison with experimental results.**
(XLSX)
